# Supplementary material for: Enhancing Breast Cancer Detection through Advanced AI-Driven Ultrasound Technology: A Comprehensive Evaluation of Vis-BUS
Source: Diagnostics (Basel). 2024 Aug 26;14(17):1867. doi: 10.3390/diagnostics14171867 (PMC11394308; doi:10.3390/diagnostics14171867)
Supplement: Supplementary file 1 [file diagnostics-14-01867-s001.zip › diagnostics-3159894-supplementary.pdf]

BARRELEYE

Beyond Ultrasound: Seeing the Unseen

Copyright © 2023 BARRELEYE All rights reserved.

PROPRIETARY AND CONFIDENTIAL

VIS-BUS (Breast Cancer) - ENHANCING WORKFLOW & ACCURACY

Workflow Convenience

- Image Recommendation Feature: Assists in comparing past images of the lesion of interest.
- Report-Generation Feature: Creates diagnostic reports based on the derived results.

Accuracy Increase

- Real-Time Ultrasound Analysis: Detects abnormal lesions through real-time ultrasound image analysis.
- BI-RADS Feature and Category Analysis: Analyzes ultrasound images to determine BI-RADS features and categories.
- Cancer Probability Score: Offers a "Cancer probability score"

Copyright ⓒ 2024 Barreleye ALL rights reserved.

PROPRIETARY AND CONFIDENTIAL

VIS-BUS (Breast Cancer) - ENHANCING WORKFLOW & ACCURACY

	KOIOS	Beamworks	VIS-BUS
Compares tumor images of the past with the current (historical tracking)	X	X	O
Generates reports for easy view	X	X	O
Detects abnormal lesions via real-time ultrasound image analysis	X	O	O
Enhances BI-RADS category accuracy	O	O	O
Offers cancer probability score	O	O	O
KFDA approval	X	Ongoing	Ongoing
FDA approval	O	Unknown	TBD

Copyright ⓒ 2024 Barreleye ALL rights reserved.

PROPRIETARY AND CONFIDENTIAL

VIS-BUS Breast Cancer Diagnostic Assistance Solution Feature (1)

Real-Time Ultrasound Analysis and
BI-RADS Feature and Category Analysis

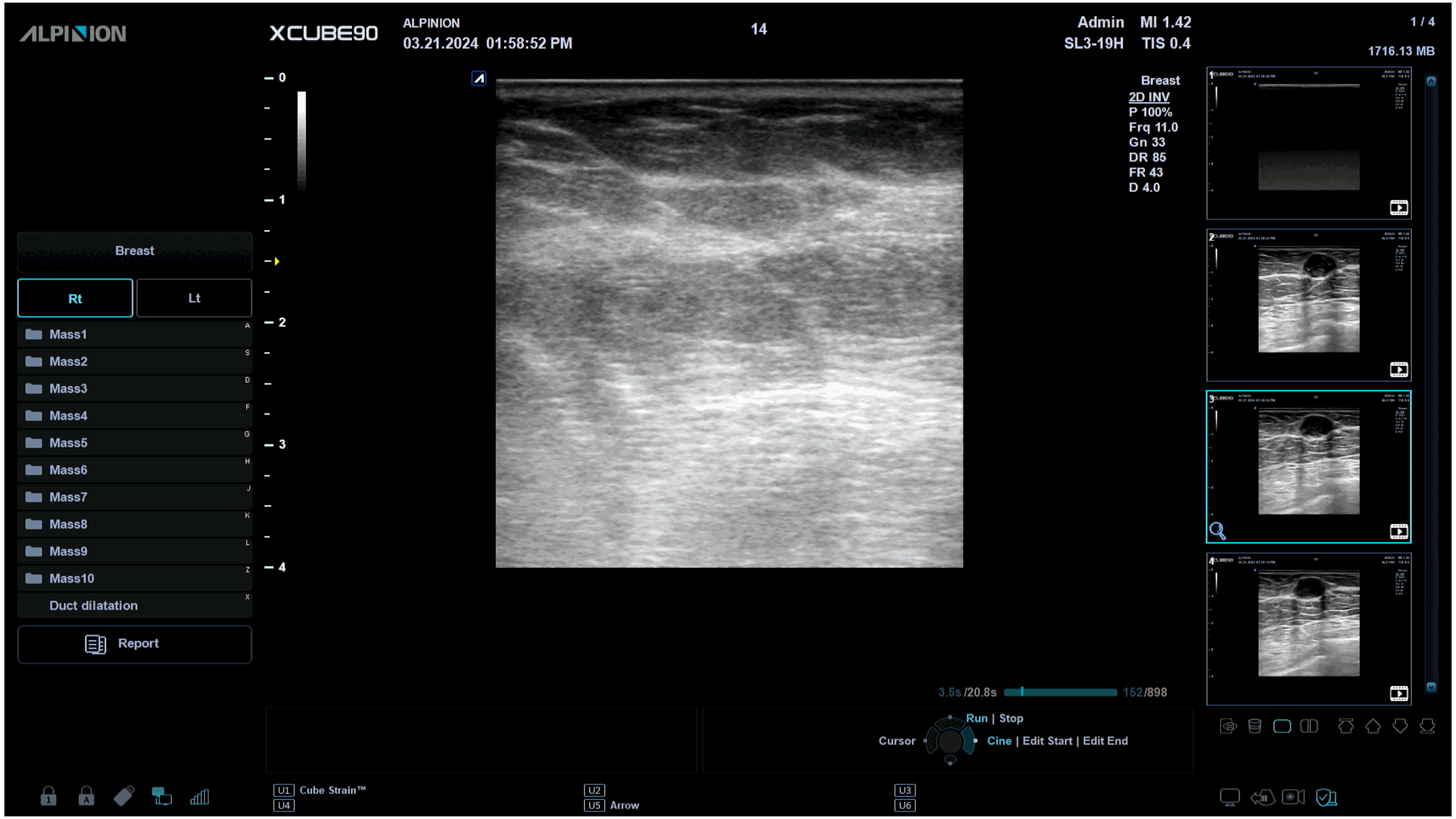

Copyright ⓒ 2024 Barreleye ALL rights reserved.

PROPRIETARY AND CONFIDENTIAL

BREAST CANCER APPLICATION

VIS-BUS Breast Cancer Diagnostic Assistance Solution Feature (2)

Cancer Probability Score

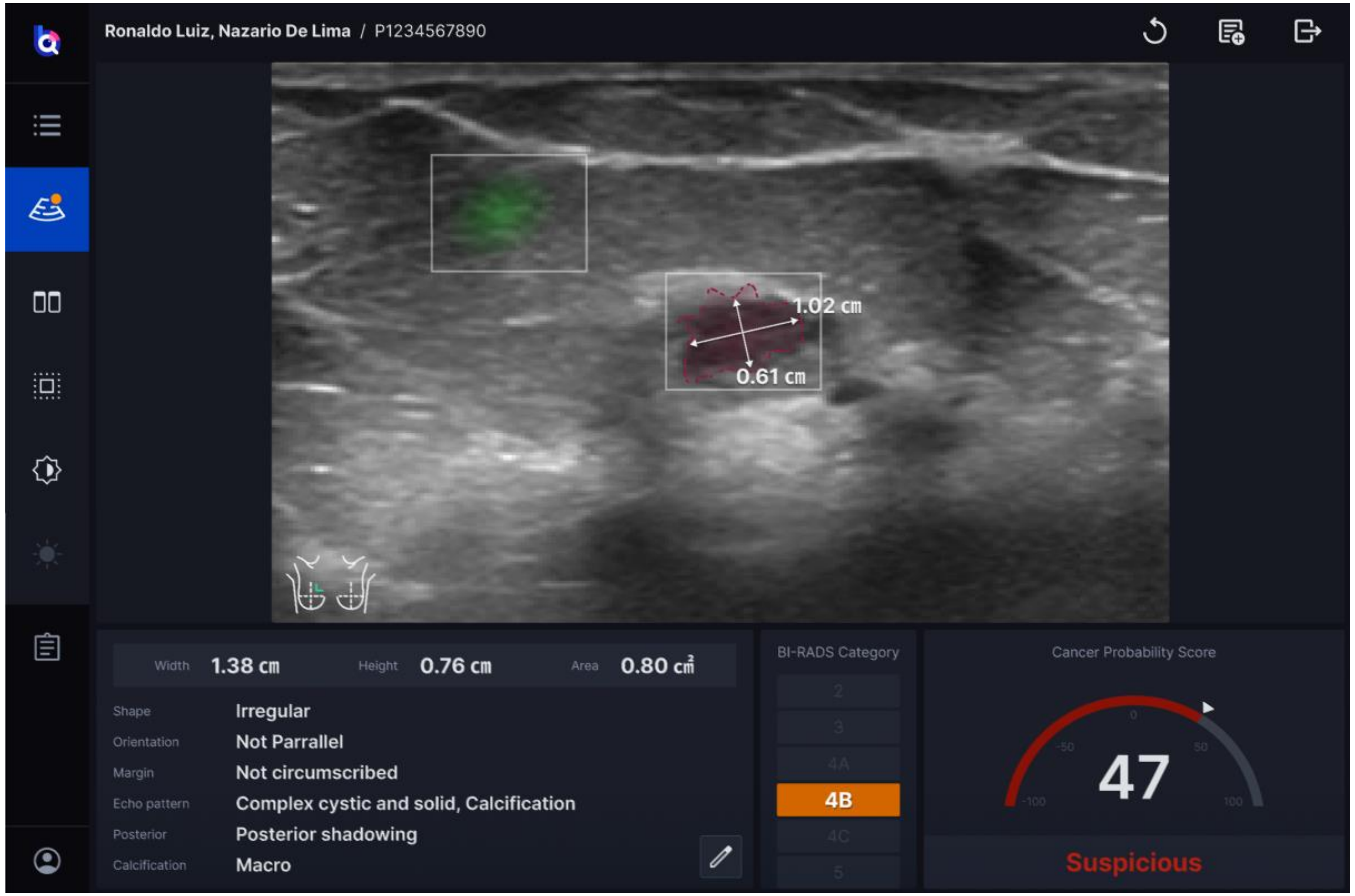

* Multiple lesion can be detected

Report Generation

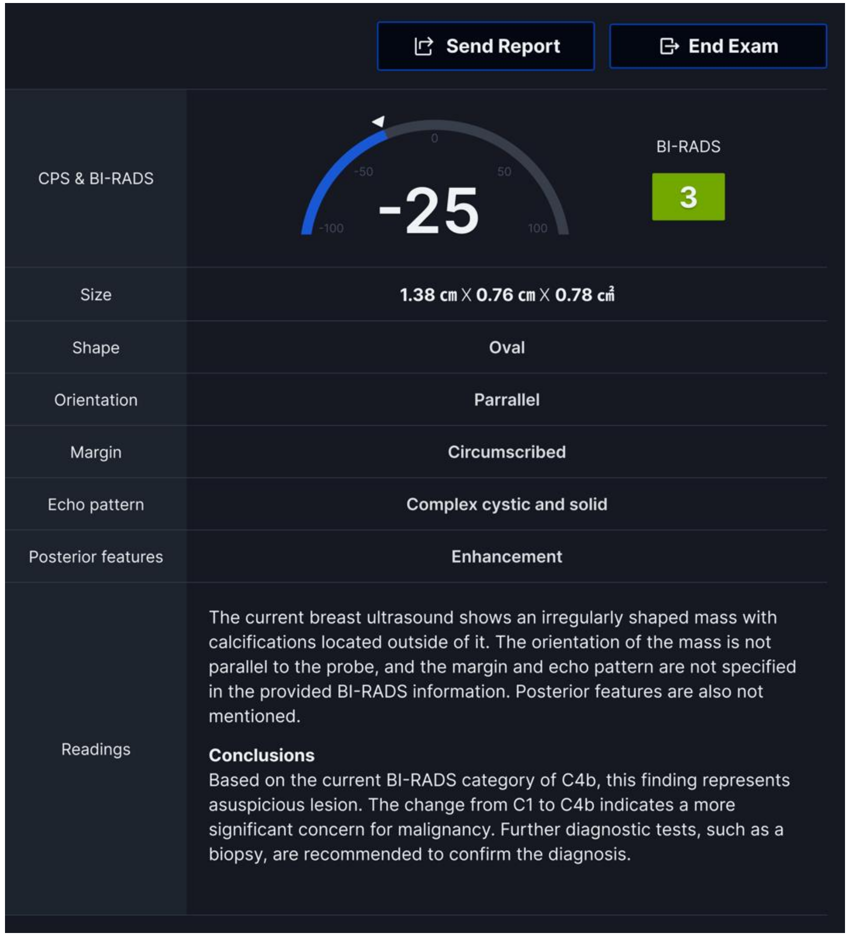

* Template of readings can be customized

Copyright ⓒ 2024 Barreleye ALL rights reserved.

PROPRIETARY AND CONFIDENTIAL

VIS-BUS Clinical Validation

Result: Pivotal clinical study approved by Korea MFDS (Ministry of Food and Drug Safety)

Diagnostic performance (AUC)

Ultrasound only: 0.89 » + Vis-BUS: 0.95

Diagnostic accuracy improved by 7%

Diagnosing time

Ultrasound only: 14 sec » + Vis-BUS: 4 sec

Diagnostic speed increased by 71%

Copyright ⓒ 2024 Barreleye ALL rights reserved.

PROPRIETARY AND CONFIDENTIAL

EASY TO USE: Direct integration with your ultrasound system

Ultrasound system　　Vis-BUS on tablet

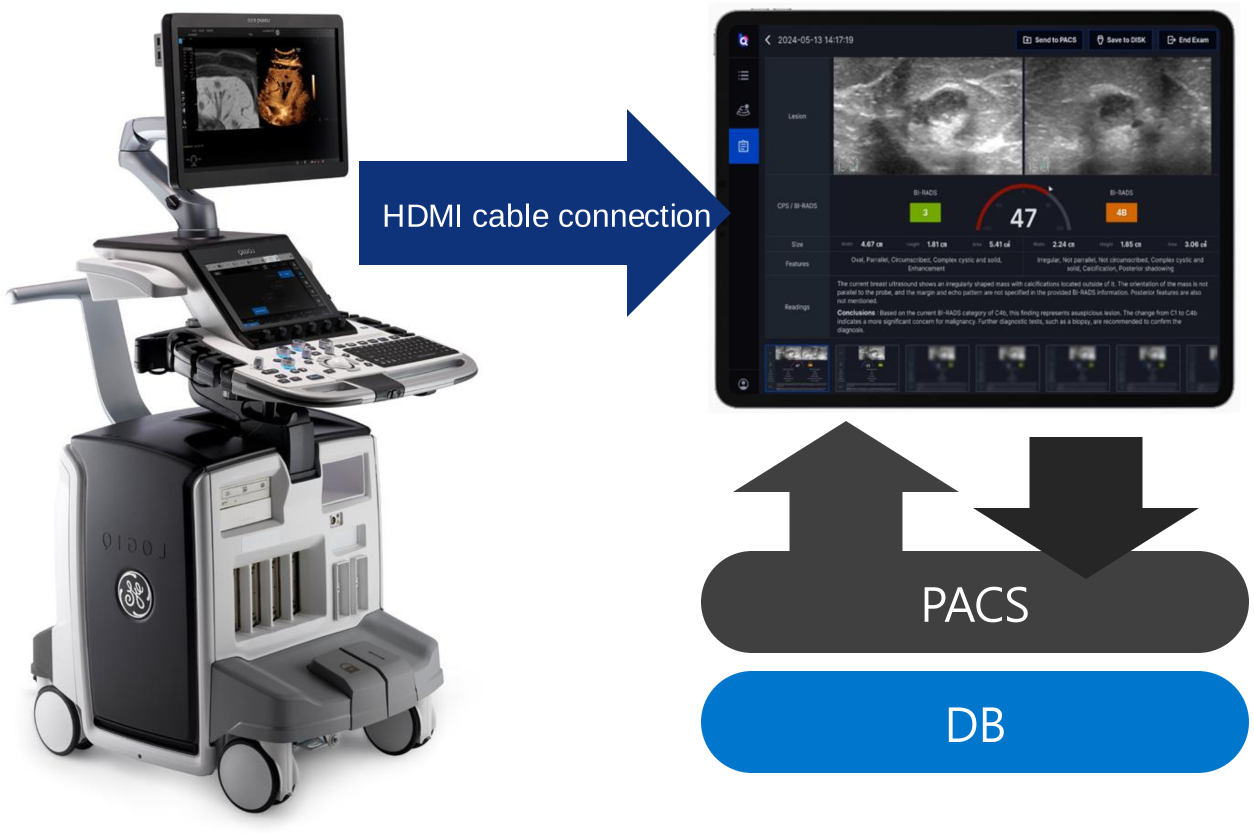

Strengths of Barreleye AI solution

- Lightweight AI easily integrated into all ultrasound devices.
- Direct integration without the need for hardware modifications.

Benefits of Barreleye AI solution

- Low initial implementation and adoption costs.
- Quick adaptation with a familiar user interface.

Copyright ⓒ 2024 Barreleye ALL rights reserved.

PROPRIETARY AND CONFIDENTIAL

Demo video

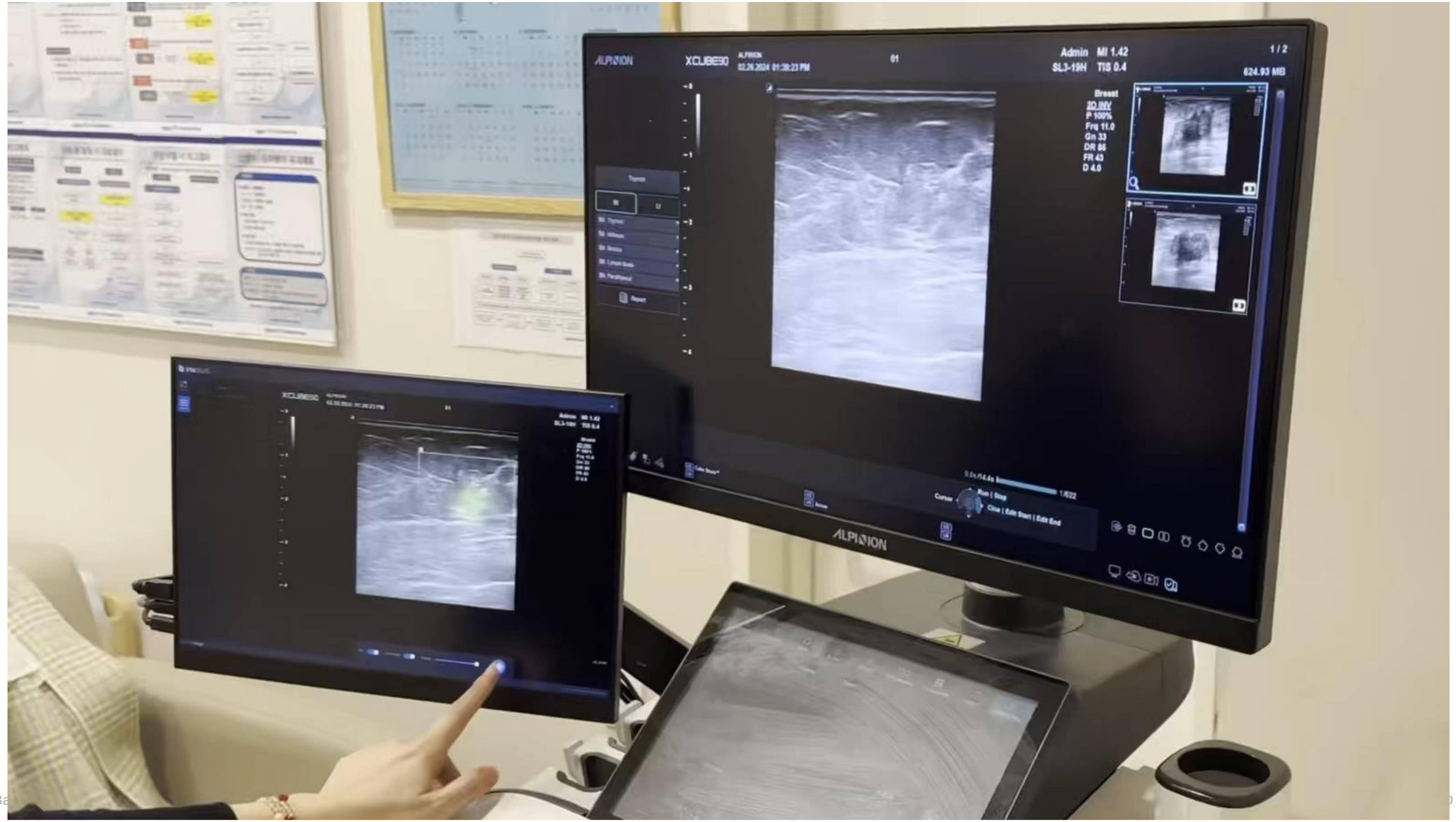

Copyright ⓒ 2024 Ba CONFIDENTIAL

Barreleye info and the team

Company History

**2016**	10	Government project (2.5M USD)
**2020**	10	Government project (6M USD)
**2021**	04	Founded Barreleye
	10	Seed Investment (650K USD)
**2022**	07	TIPS Investment (500K USD)
**2023**	04	KFDA BIOPSON (Quantitative Ultrasound System)
	05	Pre-A Investment (3M USD)

Number of employees	16
Date of establishment	2021. 04. 14
Company address	(06211) 14F Vision Tower, 312, Teheran-ro, Gangnam-gu, Seoul, Republic of Korea
Website	www.barreleye.co.kr

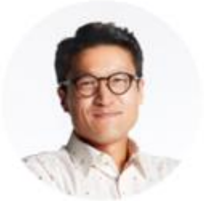

CEO / Founder

Hyeon-Min Bae, PH.D

Professor of Electrical Engineering, KAST
President of Institute for startup KAIST
Founder of Terasquare, Point2tech, and OBELAB
University of Illinois Urbana-Champaign, Ph.D

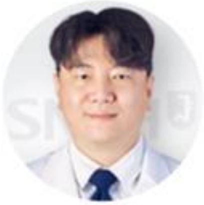

CMO / CoFounder

Hyuk-sool Kwon, MD, PH.D

Professor of Emergency Medicine, SNUBH
Founding member of International Ultrasound Conference (WINFOCUS) and SECCI

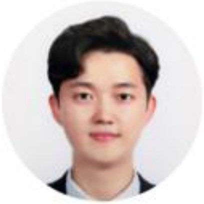

AI / CoFounder

Myeong-Gee Kim, PH.D

Specialist at Medical AI, and Ultrasound
KAIST, Ph.D

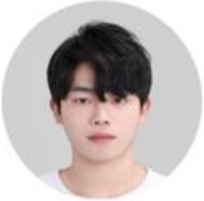

AI / CoFounder

Seok-Hwan Oh, PH.D

Specialist at Medical AI, and Ultrasound
KAIST, Ph.D

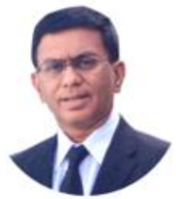

Consulting Senior Executive

Kumar Chittipeddi, PH.D

Founder-President of CEO Advisory Services
Specialist at corporate strategy, operations, and business development

Copyright © 2023 BARRELEYE All rights reserved.

PROPRIETARY AND CONFIDENTIAL
